# Causes of Polyserositis: A Systematic Review

**DOI:** 10.3390/jpm13050834

**Published:** 2023-05-15

**Authors:** Laura Elena Stoichitoiu, Georgeta Daniela Ionescu, Ingrid Neatu, Cristian Baicus

**Affiliations:** 1 Faculty of Medicine, Carol Davila University of Medicine and Pharmacy, 050474 Bucharest, Romania; cbaicus@gmail.com; 2 Department of Internal Medicine, Colentina Clinical Hospital, 020125 Bucharest, Romania; ionescu_georgiana93@yahoo.com; 3 Clinical Research Unit Bucharest, Réseau d’Epidémiology Clinique International Francophone, 020125 Bucharest, Romania; 4 Department of Dermatology, Clinical Hospital of Infectious Diseases, 800179 Galati, Romania; ingridneatu@yahoo.com

**Keywords:** polyserositis, effusion, exudate, etiology, systematic review

## Abstract

At present, polyserositis (PS) remains a challenging entity, which resides both in the fact that there is confusion regarding the terminology, and that it is still understudied. We aimed to identify the etiologies of PS, reported in adult patients. Methods: We performed a systematic review of the literature on PubMed(MEDLINE) database, using the following (MESH) terms: pleurisy/etiology, pleural effusion/etiology, pericarditis/etiology, pericardial effusion/etiology, pericardial effusion chronic, ascites/etiology, ascitic fluid/etiology, polyserositis, serositis, and serositides. Results: A total of 1979 articles were identified, dating from 1973 onwards. After screening the articles, we included 114 patients from 23 articles (one case series including 92 patients and 22 case reports) in the final report. The most common diagnosis was neoplasia (30; 26.3%), followed by autoimmune diseases (19, 16.7%) and infections (16, 12.3%). Still, in 35 cases, the etiology of PS remained unkown. Conclusion: PS is a challenging and understudied entity, which is associated with a wide range of diagnoses. However, prospective studies should be developed in order to have a clear understanding regarding its etiologies and their prevalences.

## 1. Introduction

Currently, polyserositis (PS) remains a challenging entity, which resides both in the fact that there is confusion in terminology, and that it is still understudied. Losada et al. [1] define it as the inflammation, with effusion, of different serous membranes such as the pleura, pericardium and peritoneum at the same time, while other published papers are citing the aforementioned study for the definition of polyserositis [2,3]. However, from a thorough reading, in the study published by Losada et al., the only source of the definition for PS is the Merriam-Webster dictionary, in which it is mentioned that PS is also known as Concato’s disease [4]. Nevertheless, in 1881, Concato was described for the first time in two cases of PS which were secondary to disseminated tuberculosis, while only suggesting that it may exist as a nontuberculous type of polyserositis [5]. Moreover, in order to deepen the discrepancies between the terminology, a paper titled “Idiopathic polyserositis” ascribes this terminology to the presence of transudate in pleura, pericardium and peritoneum [6]. Therefore, it is clear that even at this point, there are numerous uncertainties regarding this entity and its respective etiology.

Taking all these into account, we aimed to identify the etiologies of PS reported in adult patients.

## 2. Materials and Methods

As the prevalence can be ascertained only from case series including consecutive patients with PS, we were especially interested in these kinds of studies. In the absence of such studies, case reports (CRs) relating to PS were sought for a more comprehensive list of the etiological spectrum.

**Figure 1:**
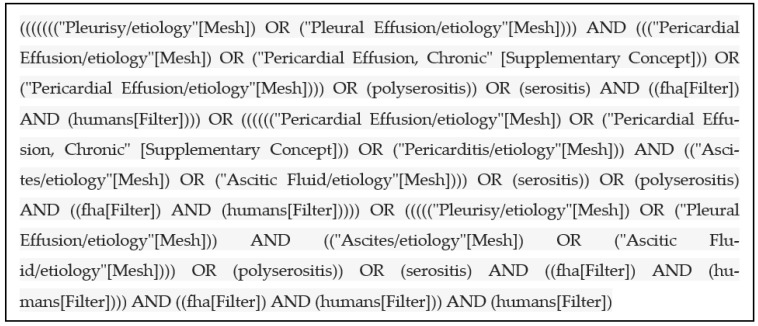
Search strategy.

A systematic review of the literature was performed in the PubMed (MEDLINE) database, using the following (MESH) terms: pleurisy/etiology, pleural effusion/etiology, pericarditis/etiology, pericardial effusion/etiology, pericardial effusion chronic, ascites/etiology, ascitic fluid/etiology, polyserositis, serositis and serositides. Figure 1 illustrates the search strategy. The question on which the research was based was: “What are the diseases that may be associated with PS?”.

An analysis was conducted on all the articles written in English and Romance languages (French, Italian, Spanish and Portuguese) between 1973 and 2020. Upon the completion of this paper, the search was done once again (June 2022) to check if any other articles meeting the inclusion criteria had been published in the meantime.

Two review authors (L.E.S., G.D.I.) independently screened all the articles based on the titles and abstracts, in order to identify the reports and studies that potentially meet the inclusion criteria. Any disagreements between the two independent reviewers were discussed with a third author (C.B.). After screening the titles and the abstracts, the full texts of all the eligible articles were retrieved and assessed for the following inclusion criteria: (1) all patients were above the age of 18 with associated inflammation and effusions of two or more serous membranes simultaneously, (2) all the effusions were confirmed by an imaging test, (3) at least one of the serous fluids was analyzed, with the result being compatible with an exudate, and (4) for case series, the presence of PS had to be an inclusion criteria. For pleural and pericardial fluids, Light’s criteria were accepted as proof [7]. For ascites, besides Light’s criteria, a serum-ascites albumin gradient less than 1.1 g/dl was accepted, but with the exclusion of the nephrotic syndrome in the latter case.

The exclusion criteria included any of the following: (1) papers without evidence of PS, (2) papers involving people under the age of 18, (3) papers not involving humans, (4) papers not mentioning if the effusions were transudates or exudates, (5) papers not mentioning which criteria, or if any criteria were fulfilled in order to affirm that the effusions were exudates, (6) papers labeling the effusions as exudates according to the tests performed on previous presentations, unrelated to the actual acute event, (7) papers labeling the effusions as PS, without acknowledging if the effusions were present in at least two cavities at the same time, (8) narrative reviews and systematic reviews, (9) study protocols, (10) papers which did not have the full text available, and (11) publications in languages other than those mentioned above. 

## 3. Results

**Figure 2:**
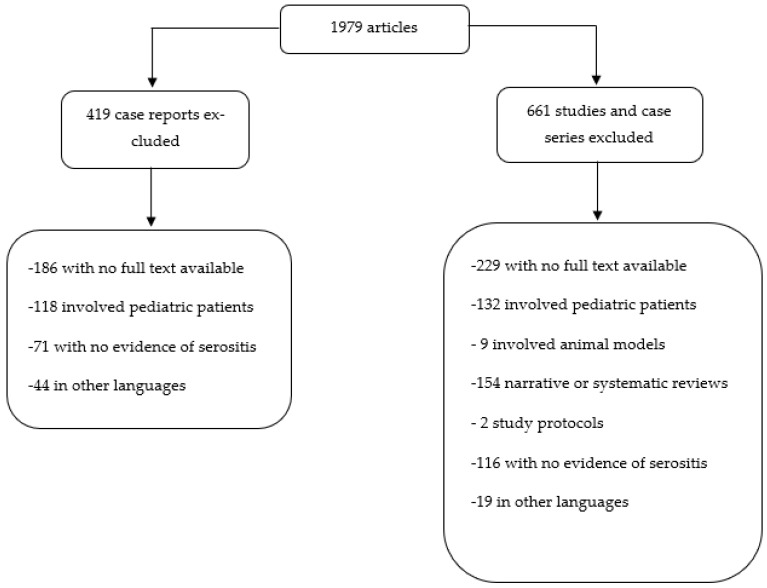
Articles excluded based on the title and abstract.

A total of 1979 articles were identified. At first, the papers were screened based on the titles and abstracts and a total of 1080 articles were excluded, the in extenso criteria which are justifying our decisions being illustrated in Figure 2.

**Figure 3:**
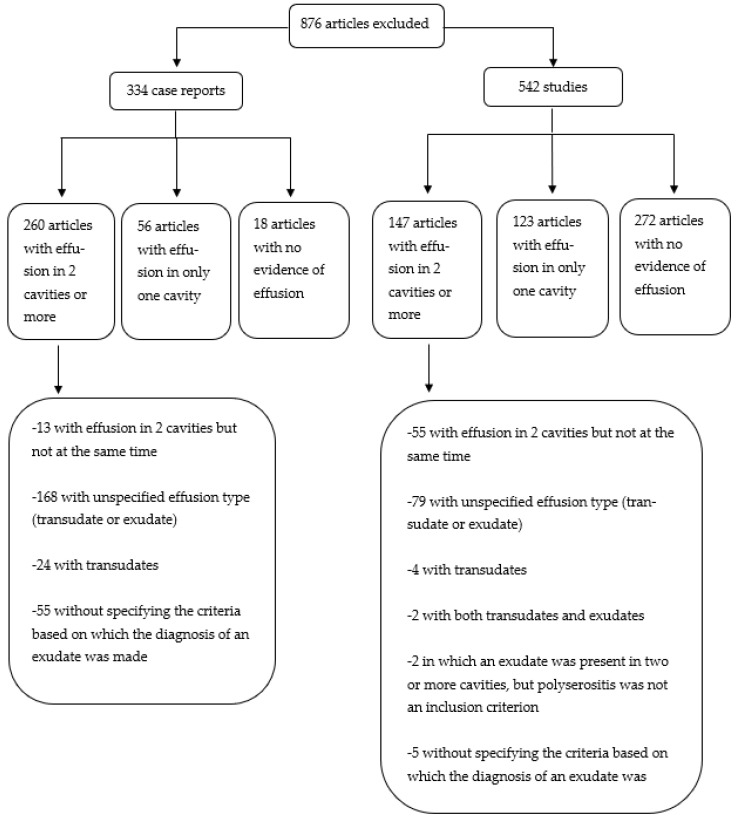
Articles excluded based on full-text analysis.

After analyzing the full text of the remaining 899 publications, another 876 papers were discarded and not included in this research. The exclusion criteria are further detailed in Figure 3. The final report included 23 articles (one case series of 92 patients and 22 case reports) which encompassed all the inclusion criteria.

Among the 114 human subjects with PS, 67 (58.8%) were females. The mean age of the patients was 59 years old.

Ten patients (8.8%) had a history of autoimmune diseases [1,8]. Twenty patients (17.5%) had a known previous history of cancer. Hematological neoplasia (7; 35%) and lung cancer (7; 35%), which were identified in an equal number of patients, were the most common types [1,9,10,11,12]. Two of the six remainders had a previous history of breast or ovarian carcinoma [13,14], while in Losada’s study the other types of neoplasia were not mentioned [1]. However, only four patients had any evidence of active cancer [1].

The most frequently encountered symptoms were dyspnea (53; 46.5%) and thoracic pain (32; 28%). Other less frequent symptoms were fever, cough, arthralgia, weight loss, cutaneous lesions, edema, abdominal pain and malaise.

Regarding the etiology, the most common diagnosis was neoplasia (30; 26.3%), which occurred in about one quarter of the cases, followed by autoimmune diseases (19; 16.7%) and infections (16; 12.2%). However, the etiology of PS remained unknown in 35 cases (30.7%) [1,15].

Other causes were identified in 14 cases (12.3%). One patient, with relapsed myelodysplastic syndrome after allogenic hematopoietic stem cell transplantation, was diagnosed with chronic graft-versus-host-disease-associated serositis (bilateral pleural effusion and ascites), which improved after a second allogenic hematopoietic stem cell transplantation [10], and one woman with ovarian hyperstimulation syndrome developed a large amount of bilateral pleural effusion and a slight amount of ascites, with improvement after drainage [16]. In another CR, the first systemic inflammatory reaction was presented to the 13-valent pneumococcal conjugate vaccine in a patient without a previous autoimmune disease, who progressively developed polyarthralgia, myalgias, and finally marked serositis of the pleura and pericardium [14]. Besides the aforementioned conditions, PS was also due to Meigs Syndrome (two cases) [12,17], one pseudo-Meigs syndrome associated to ovary leiomyoma [18], hypothyroidism (one case) [19], drug-induced lupus erythematosus secondary to trimethoprim/sulfamethoxazole (one case) [20] and dasatinib (one case) [9]. Additionally, one CR with ergotamine-induced pleural and pericardial effusion was identified. Ergotamine ingestion was considered the cause of the exudative effusion after the exclusion of common etiologies and after achieving fluid remission with drug withdrawal [13]. In the documented CRs, all patients with a toxic origin of PS had pleural and pericardial involvement (3; 100%). Additionally, in the study of Losada et al., there were also four cases of PS ascribed to other causes, but without any further clarifications in regard to the etiology [1]. A more detailed picture regarding the characteristics of the patients identified from CRs is illustrated in Table 1.

Among the 19 patients with a final diagnosis of an autoimmune disease, the most common condition was systemic lupus erythematosus (SLE) (11; 58%) [1,21,22,23]. The remaining subjects were diagnosed with Adult Still’s disease (3; 15.8%), rheumatoid arthritis (RA) (2; 10.5%), dermatomyositis (1; 5.3%), IgG4-related disease (1; 5.3%) and antisynthetase syndrome (1; 4.5%) [1,8,24,25,26].

Antinuclear antibodies (ANAs) were studied in 78 (68.5%), with a total of 25 (21.9%) positive results. Unfortunately, the data from the case series did not permit the calculation of sensitivity, specificity or false positive/negative rates [1]. However, in Losada et al., ANA positivity was associated with an autoimmune disease as the etiology for PS (OR 14.3 (2.8–73.3); *p* = 0.004) [1]. Anti-cytoplasmic antibodies (ANCAs) were analyzed in 47 (41.2%) subjects, and in 9 (19.1%) of them, they were positive, but without a final diagnosis of ANCA-associated vasculitis. Anti-DNA antibodies were studied in 62 patients (54.4%), with only 5 (8%) positive results. Rheumatoid factor was analyzed in 8 patients (36.4%), all from the 22 case reports included, with only 3 patients having the test positive [14,22,26]; however, only 1 of them had a final diagnosis of RA, with associated ascites, pericardial and pleural effusion, in whom pleural biopsy confirmed chronic inflammation and fibrosis. The ascites, pleural and pericardial fluid were considered extraarticular manifestations of RA, and did not recur after immunosuppressive therapy [26]. The other patients with a positive rheumatoid factor were diagnosed with PS induced by the 13-valent pneumococcal conjugated vaccine [14] and SLE [22], while in the study of Losada et al., nothing is mentioned about the positivity of the rheumatoid factor, even though they enrolled one patient with RA [1]. Additionally, there were two CRs in which a normal autoantibody screen [27] and unremarkable autoantibodies [12] are mentioned, but without any further details.

Sixteen patients with infectious diseases developed PS. In 2 of the 22 CRs included, two patients with infectious diseases were described, and both of them had an immunosuppressive state. One patient, with orthotopic liver transplantation for decompensated cirrhosis due to hepatitis C virus and immunosuppressive treatment with tacrolimus, developed exudative serositis. Although no Mycobacterium tuberculosis pathogen was observed after microscopic analysis and cultures were negative, a positive polymerase chain reaction assay was obtained and Mycobacterium tuberculosis infection was considered the etiology of PS [28]. The second case of infectious disease is represented by disseminated cryptococcosis in a patient with myelodysplastic syndrome and steroid therapy for organizing pneumonia [11]. Losada et al. described 14 patients with infectious diseases, with the following pathogens being incriminated as causative: Coxsackie virus (six cases), Cytomegalovirus (one case), Epstein–Barr virus (one case), Coxiella burnetii (one case) and Mycobacterium tuberculosis (one case). The remaining four cases were ascribed to “other” pathogens, without any further clarifications regarding the causative agent [1].

For the disposition of the effusions, almost all patients (113; 99.1%) had pleural involvement. Most subjects described in the CRs had bilateral pleural effusions (15 out of 22; 68.2%), while in the study of Losada et al., it is not mentioned if the involvement was unilateral or bilateral; additionally, it is not mentioned by which imagistic means the presence of the effusions was ascertained [1]. Regarding the CRs, almost all patients had a computed tomography scan (CT) of the thorax (18; 81.8%); a chest X-ray was performed in nine (40.9%), while only two patients (9.1%) had a pleural ultrasound.

Pleural fluid was analyzed in 93 (82.3%) patients, and in nearly half (41; 44.1%), a lymphocytic exudate was revealed.

Overall, adenosine deaminase (ADA) levels were increased in 9 cases with lymphocytic exudate (22%), and LDH levels were increased in 26 pleural effusions (26; 28%). In the Losada et al. study, an increased ADA level was associated with a final diagnosis of an autoimmune disease, and, surprisingly, not with an infectious disease, while an increased LDH level was associated with a diagnosis of neoplasia [1]. Pleural fluid cultures were analyzed in 77 (82.8%) patients, with only 2 (2.6%) of them being positive: one for Mycobacterium tuberculosis and one for Streptococcus pneumoniae. Pleural biopsies were performed in only four patients (3.5%) identified from the CRs [13,24,26,28]. In neither case did the pleural biopsy represent the decisive test for the final diagnosis.

Ninety-three patients (81.6%) had pericardial effusions and 29 patients (25.4%) had ascites. Pericardial fluid was analyzed in 20 patients (21.5%), and in 8 cases (40%), a hematic pericardial fluid was identified [1,14]. Only one pericardial biopsy was performed in a patient with bloody pericardial effusion and a final diagnosis of PS induced by the 13-valent pneumococcal conjugated vaccine [14]; all cultures were negative.

Among the patients with ascites, a paracentesis was performed in 18 cases (62.1%); all cultures were negative. Four of them (22.2%) had a cytology conclusive of malignancy. Only one peritoneal biopsy was performed in a patient with a final diagnosis of Mycobacterium tuberculosis infection [28]. One patient presented chylous ascites, with markedly elevated triglycerides at 568 mg/dl, which turned out to be due to SLE [21]. In the 22 CRs included, 13 patients had ascites. An abdominal ultrasound was performed in 4 patients from the total of 22 CRs (18.2%), while a CT scan of the abdomen was performed in 8 (36.4%) cases.

Among all 114 subjects, 85 patients (74.6%) presented fluid in the pleura and the pericardium at the same time, and 21 patients (18.4%) had pleural and peritoneal involvement, while only 1 patient (0.9%) presented with pericardium and peritoneal effusions. PS consisting of fluid accumulation in all three cavities (pleural, pericardial and peritoneal) was documented in seven (6.1%) cases. The association between the effusion disposition and the final diagnosis is illustrated in Table 2. For the prevalence of etiologies, given the fact that these can be ascertained solely from case series including consecutive patients, we were able to calculate them only from the Losada et al. study; idiopathic PS was encountered most frequently, in 36.95% of cases, followed by neoplasia (30.43%), infectious diseases (15.21%) and autoimmune diseases (13.04%). Other causes were identified in 4.34% of cases.

The fluid was therapeutically drained in 10 patients (45.5%); all of them were identified from the CRs. Most patients were successfully treated, and PS resolved in 77 patients (67.5%). PS recurred in 9 cases (7.9%) and persisted in 12 (10.5%). Twenty-seven patients died during hospitalization or the follow-up period: 24 of them had a final diagnosis of neoplasia [1,29] and 2 of them died due to infectious complications [1,14]. In one patient, the cause of death, nor the final diagnosis were mentioned [1].

**Table 1:** Patients with polyserositis included in our research (22 case reports).

Ref	G	Diagnosis	Serosal Involvement	Means of Diagnosis	Differential Diagnosis	Treatment	Follow-Up
[24]	F	Adult Still’s disease	Pleura (bilateral)–pericardium–peritoneum	Positive Cush and Yamaguchi criteria, bone marrow biopsy, axillary lymph node biopsy, exploratory laparoscopy	Infectious diseases, autoimmune diseases, neoplastic processes	CS, Cyclophosphamide, paracentesis	Successfully treated, no recurrence for four years
[21]	F	SLE	Pleura (left)–peritoneum	SLICC criteria, bone marrow biopsy, fluid analysis, exploratory laparoscopy	Other autoimmune diseases, tuberculosis, neoplasia	CS, mycophenolate mofetil, granulocyte colony stimulating factor	At 6 months, residual minimal left-sided pleural effusion
[25]	F	Dermatomyositis	Pleura (bilateral)–peritoneum	Typical skin manifestations, elevated muscle enzymes, muscle weakness, and myogenic pattern on electromyography, lower limbs MRI	Autoimmune diseases, infectious diseases, tuberculosis	CS, Cyclophosphamide, Tacrolimus, intravenous immunoglobulins	No recurrence of pleural effusion or ascites after 70 days
[26]	M	Rheumatoid arthritis	Pleura (bilateral)–pericardium–peritoneum	Clinical manifestations, ultrasound, pleural biopsy, subcutaneous nodules biopsies, imagistic investigations, fluid analysis	Autoimmune diseases, infectious diseases, neoplasia	CS, methotrexate, NSAIDs	Disease remission
[22]	M	SLE	Pleura (bilateral)–peritoneum	Clinical manifestations, serology, fluid analysis	Other autoimmune diseases, hypothyroidism	CS	Remission
[8]	M	Adult Still’s disease	Pleura (bilateral)–pericardium	Yamaguchi criteria exclusion of autoimmune diseases	Autoimmune disease	CS, intravenous immunoglobulins	Remission
[23]	M	SLE	Pleura (bilateral)–pericardium	Clinical manifestations, serology, imagistic investigations, fluid analysis	Heart failure, other autoimmune diseases, infectious diseases, neoplasia	CS, mycophenolate mofetil	Serositis improvement
[29]	F	Acute myelogenous leukemia with myeloid sarcoma	Pleura (bilateral)–pericardium	Axillary lymph node biopsy, bone marrow biopsy, flow cytometric and cytogenetic analysis of both effusions	Other types of hematological neoplasms	Antibiotics, antifungals, cytostatic regimens	Death after 69 days
[18]	F	Pseudo-Meigs Syndrome associated to ovary leiomyoma	Pleura (right)–peritoneum	Fluid analysis, CT scan, omental biopsy, histopathological analysis of the mass arising from the left ovary	Infectious diseases, heart failure, constrictive pericarditis, cirrhosis, hypothyroidism, neoplastic processes	Surgery (histerectomy and bilateral salpingo-oophorectomy), drainage of the pleural effusion	Complete resolution of PS
[27]	F	Primary effusion lymphoma	Pleura (bilateral)–pericardium	Imagistic investigations, effusion analysis (including cytology, immunohistochemistry, and FISH analysis)	Autoimmune diseases, hypothyroidism, infectious diseases	Pleural drainage, pericardial effusion resolved with the first pleural drainage	One unilateral recurrence which resolved with drainage, and without any additional recurrence
[28]	M	Mycobacterium tuberculosis infection after orthotopic liver transplantation	Pleura (unilateral)–peritoneum	Fluid analysis with positive Mycobacterium tuberculosis PCR, but negative cultures	Other bacterial and viral infections	Isoniazid, ethambutol, streptomycin, ciprofloxacin	No recurrence of serositis
[17]	F	Meigs syndrome	Pleura (right)–peritoneum	Fluid analysis with elevated CA-125, exploratory laparotomy, histopathological analysis of the mass arising from the left ovary	nd	Pleural abrasion with talc pleurodesis; histerectomy and bilateral salpingo-oophorectomy	No recurrence of pleural effusion 12 weeks after surgery
[12]	F	Meigs syndrome	Pleura (bilateral)–pericardium–peritoneum	CT scan, fluid analysis, histopathological analysis of the mass arising from one ovary	Neoplastic processes, infectious diseases	Right pleural drainage, histerectomy and bilateral salpingo-oophorectomy	No relapse 8 months after surgery
[9]	M	Drug-induced lupus to dasatinib	Pleura (bilateral)–pericardium	Presence of autoantibodies (ANA, anti-RNP/Sm) which became undetectable after switching to Nilotinib, natural evolution (remission of effusion with dasatinib discontinuation, and reoccurrence with dasatinib resumption	Malignancy, other autoimmune diseases, tuberculosis	CS, mycophenolate mofetil, diuretics, pleural drainage	Remission
[19]	F	Hypothyroidism	Pleura (left)–pericardium	Low levels of fT4 and pathologically elevated TSH during continued high-dose methimazole, exclusion of other causes	Neoplastic, infectious, and autoimmune diseases, heart failure	Discontinuation of methimasole dose, short course of levothyroxine, diuretics	Nearly remission of PS at 3 months
[20]	F	Drug-induced lupus secondary to trimethoprim/sulfamethoxazole	Pleura (bilateral)–pericardium	Recent administration of trimethoprim/sulfamethoxazole, fluid analysis, serology (anti-histone antibody positivity)	Autoimmune and infectious diseases	Short course of CS; pericardiocentesis (Cardiac tamponade)	PS remission 6 months after discharge
[13]	F	Ergotamine-induced serositis	Pleura (bilateral)–pericardium	Prolonged use of ergotamine and caffeine, exclusion of other causes, fluid analysis, pleural biopsy	Malignancy, autoimmune diseases, infectious diseases (including tuberculosis)	NSAIDs, colchicine, stopping ergotamine	PS remission4 months after discharge
[16]	F	Ovarian hyperstimulation syndrome	Pleura (right)–peritoneum	History (recent ovarian hyper stimulation), abdominal ultrasound (enlarged ovaries with multiple follicular cysts), pleural effusion analysis	nd	Pleural drainage	Remission
[10]	M	Graft versus host disease after allogenic hematopoietic stem cell transplantation	Pleura (bilateral)–peritoneum	Exclusion of other causes, associated bronchiolitis obliterans	Malignancy, infectious diseases	Inhaled CS, antibiotics	Remission of PS
[14]	F	Polyserositis induced by the 13-valent pneumococcal conjugated vaccine	Pleura (left)–pericardial	Exclusion of other causes, case history (temporal association with recent administration of vaccine), presence of a persistent local reaction to the 13-valent pneumococcal conjugated vaccine	Autoimmune diseases, infectious diseases, malignancy, autoimmune/autoinflammatory syndrome induced by adjuvants (ASIA)	CS, pericardial and pleural drainage	No recurrence for one and a half year since discharge
[15]	M	Idiopathic (labeled by the authors as “inflammatory ascites”)	Pleura (not mentioned if unilateral or bilateral)–pericardium–ascites	Exclusion of all other causes	Neoplasia, hypothyroidism, infectious diseases, autoimmune diseases	CS, paracentesis	Complete resolution after one month
[11]	M	Disseminated cryptococcosis	Pleura (right)–peritoneum	Blood cultures, serum, pleural, peritoneal and cerebrospinal fluid positive for cryptococcal antigen; transbronchial lung biopsy from pulmonary lesions	nd	Antifungals, antibiotics (for later superimposed bacterial infection)	Death

G—gender; CS—corticosteroids; SLE—systemic lupus erithematosus; SLICC—Systemic Lupus International Collaborating Clinics; NSAIDs—non-steroidal anti-inflammatory drugs; nd—not described; CT—computed tomography; MRI—magnetic resonance imaging; FISH—fluorescence in situ hybridization; PCR—polymerase chain reaction test.

**Table 2:** Etiological diagnosis according to serosal involvement (all 114 patients).

Serosal Involvement	Etiology	Number
Pleura–pericardium (*n* = 84)	Autoimmune	13
	SLE	8
	Adult Still’s disease	2
	Sjogren’s syndrome	1
	Ankylosing spondylitis	1
	IgG4 related disease	1
	Neoplasm	19
	Lung adenocarcinoma	9
	Lymphoma	4
	Leukemia	1
	Other lung neoplasm	2
	Other	3
	Infectious	14
	Coxsackie virus	6
	Cytomegalovirus	1
	Epstein-Barr virus	1
	Coxiella burnetti	1
	Mycobacterium tuberculosis	1
	Other	4
	Idiopathic	32
	Other	6
Pleura–ascites (*n* = 22)	Neoplasm	9
	Ovarian adenocarcinoma	2
	Gastric adenocarcinoma	2
	Lymphoma	1
	Other	4
	Autoimmune	4
	SLE	3
	Dermatomyositis	1
	Infectious	2
	Mycobacterium tuberculosis	1
	Cryptococcus neoformans	1
	Other	7
Pericardium-ascites (*n* = 1)	Neoplasm	1
	Pancreatic adenocarcinoma	1
Pleura–pericardium–ascites (*n* = 7)	Autoimmune	2
	Adult Still’ s disease	1
	Rheumathoid arthritis	1
	Neoplasm	1
	Unknown origin adenocarcinoma	1
	Other	1
	Idiophatic	3

## 4. Discussion

This systematic review aimed to assess the current literature in order to illustrate the etiology of PS. Patients (114) from one case series involving 92 patients and 22 CRs were included in the final analysis. The most common diagnosis was neoplasia (30; 26.3%), followed by autoimmune diseases (19, 16.7%) and infections (16, 12.3%). However, the etiology of PS remained unknown in 35 cases (30.7%), 34 of them coming from Losada’s study [1] and only 1 from the CRs. This is probably due to the fact that there is a propensity toward publishing CRs with an identified etiology and unique features rather than cases with an unknown cause.

Recently, it has been proposed that an association between pleural effusion and acute or recurrent pericarditis exists, being framed as a special subset of PS and having striking resemblances with autoinflammatory diseases, both seeming to be mediated by interleukin-1 overproduction [30,31]. Therefore, it is worth drawing attention to the fact that after excluding the more frequent causes for PS through a thorough work-up, autoinflammatory diseases should also be considered, especially when facing elevated levels of C-reactive protein and neutrophil leukocytosis in the presence of pleuro-pericardial effusions [30,31].

As mentioned previously, we encountered confusion when using this terminology. Until now, we did not find any chapter in a book or online medical database to address PS as a whole; instead, pleurisy/pleural effusion, pericarditis/pericardial effusion and ascites/peritonitis are treated every time as separate entities. As consequence, this leads to a lack of knowledge regarding the definition, prevalence and etiology of PS. As we pointed out previously, there is a paucity of data regarding the definition. In addition to Losada et al.’s study and Merriam-Webster dictionary, in *Dorland’s Illustrated Medical Dictionary*, PS is defined as a “general inflammation of serous membranes with serous effusions”, with another reference to Concato’s disease; however, neither which serous membranes should be involved, nor any diagnostic criteria is mentioned [32]. However, in the same dictionary, serositis is defined as the “inflammation of a serous membrane”, while the term polyserositis is also ascribed to multiple serositis. This time, the presence of an associated serous effusion is not mandatory. These discrepancies were also noted during our research. We encountered many papers in which serositis was mentioned, but without acknowledging if any effusion was present at all, or in which the term serositis and polyserositis were used interchangeably, in the absence of any serous effusion, most frequently associated with SLE or familial Mediterranean fever [33,34,35]. Moreover, when it comes to SLE, it is well known that the presence of serositis, including pleural and/or pericardial effusion, is encountered in all the classification criteria for SLE [36,37,38]. While for the presence of pericardial effusion it is clearly mentioned in the SLICC criteria [36] that other causes must be excluded, in the 2019 ACR classification criteria, the only reference made is that it should be imaging evidence of pleural or pericardial effusion, or both [36]. Even though nothing is clearly mentioned about the mandatory presence of an exudate, in order to fulfill the respective criterion, it is thought as common sense to exclude other causes of serositis, as pointed out by other papers [39,40,41]. This is of further extreme importance, due to the fact that effusions caused by active serositis have a different treatment and imply a different prognosis when compared with transudates or exudates due to other causes. In spite of this, during the screening process for this systematic review, we encountered CRs with effusions labeled as SLE-associated serositis and counted as a criterion, even though the patient had nephrotic or nephritic syndrome (and as consequence hypoalbuminemia), and in the absence of any specification regarding the evaluation of the effusion [42,43]. Therefore, especially in SLE patients in whom lupus nephritis (and in many cases hypoalbuminemia) is encountered, the necessity to rule out other causes should be reiterated and strengthened. This propensity toward ascribing the term “exudative” to an effusion was also encountered in other cases in which there were either other clear causes for the presence of an effusion and in the absence of any exudate criteria whatsoever, acute renal failure with anuria in a patient with sepsis and generalized edema [44], or in patients in whom the fluid was analyzed and proven to actually be a transudate [45]. Therefore, it is clear that there is not only a paucity of data regarding PS, but also that the actual information is, in many cases, of low quality. As a consequence, we consider that it is mandatory to standardize the definition of PS, which implies to establish the serous membranes which may be involved (at least two out of the following three: pleura, pericardium and peritoneum), and make a clear distinction between PS and anasarca. While in the latter, the presence of a transudate is implied [46], in the former, it should be emphasized that in order to make the diagnosis, the presence of an exudate is mandatory, which should be certified through Light’s criteria for pleural effusions, or serum–ascites albumin gradient criteria for ascites after the exclusion of the nephrotic syndrome. Therefore, the term PS should be reserved for diseases which involve an inflammatory process of the serous membranes, while anasarca should be used for afflictions associated with transudates, such as congestive heart failure, nephrotic syndrome or cirrhosis. Regarding pericardial effusions, even though Light’s criteria [7] are commonly used to differentiate between exudates and transudates, which was also the case for the papers included in our final analysis, there is growing evidence [47,48] that their applications misclassify normal pericardial fluids as exudates. This is due to the fact that, when compared to pleural effusion, pericardial fluid is characterized by higher levels of protein, albumin, nucleated cells (especially mesothelial cells) and very high levels of LDH. Consequently, Light’s criteria should be used cautiously for pericardial fluid analysis, while further research in this area should be made in order to establish new rules that could accurately discriminate between exudates and transudates. The main limitation of our study resides in the lack of studies (prospective or retrospective) that have PS as an inclusion criterion, and also the lack of CRs regarding this topic. In order to gain knowledge regarding the etiology of PS and its prevalence, we consider that a clear distinction should be made between serosal inflammation with and without effusion, and that there should be different terms for each situation. As an argument to stand for our opinion, we consider that the diagnosis of pleuritis, pericarditis and peritonitis, in the absence of fluid formation, is unpardonably related to subjectivism, given the interobserver accordance variability. Therefore, the inclusion of such patients in prospective studies could be biased due to many variables, including physicians experience, which may lead to erroneous data reporting.

## 5. Conclusions

PS is a challenging and understudied entity which is associated with a wide range of diagnoses, most frequently malignancy, autoimmune and infectious diseases. Moreover, every patient should have the presence of an exudate confirmed before establishing the diagnosis of PS. It is important to make efforts to standardize the definition of PS, and further prospective studies should be developed in order to have a clear view regarding its etiology and prevalence.

## Data Availability

Not applicable.
